# Interpersonal stress generation among young adolescents: vulnerable and resilient interpersonal behaviors and the generation of negative and positive interpersonal events

**DOI:** 10.3389/fpsyg.2023.1246927

**Published:** 2023-11-07

**Authors:** Yuji Kuroda

**Affiliations:** Center for Arts and Sciences, Fukui Prefectural University, Fukui, Japan

**Keywords:** stress generation, negative interpersonal events, positive interpersonal events, social withdrawal, excessive reassurance-seeking, prosocial behavior, vulnerability, resiliency

## Abstract

**Background:**

Theoretical and empirical studies on stress generation suggest four event generation processes: (1) vulnerability factors predict more negative interpersonal events; (2) vulnerability factors predict fewer positive interpersonal events; (3) resiliency factors predict fewer negative interpersonal events; and (4) resiliency factors predict more positive interpersonal events. However, few studies have examined these four processes simultaneously within a single analytic model. Therefore, it is unclear whether vulnerability and resiliency factors make unique and differential contributions to the occurrences of negative and positive interpersonal events.

**General objectives:**

This study aimed to fill this important gap by examining whether social withdrawal and excessive reassurance-seeking (vulnerable interpersonal behaviors) and prosocial behaviors (a resilient interpersonal behavior) uniquely and differentially predict the occurrences of negative and positive peer events among young adolescents. This study also examined the sex differences in these relationships.

**Methods:**

One hundred and ninety-eight students (109 girls) were recruited from a public middle school in Japan. A multiple-group path analysis was conducted to examine possible sex differences.

**Results:**

Social withdrawal uniquely predicted more negative peer events for boys and fewer positive peer events for boys and girls. Excessive reassurance-seeking uniquely predicted both more negative peer events and more positive peer events for boys and girls. Prosocial behavior uniquely predicted more positive peer events for boys and girls.

**Conclusion:**

This study underscores the unique and differential roles of vulnerable and resilient interpersonal behaviors in predicting negative and positive peer events among young adolescents. These findings not only advance our understanding of stress generation processes but also have broader implications for adolescent development and well-being.

## 1. Introduction

Previous studies have found that negative and positive life events are important risk and protective factors for depression, respectively (Hammen, 2005; Ibarra-Rovillard and Kuiper, 2011; Vrshek-Schallhorn et al., 2020). The stress generation model (Hammen, 1991, 2006) posits that individuals are not randomly exposed to their life experiences and environments, but actively create them, suggesting that they contribute to the occurrence of their life events. Identifying what and how factors contribute to the occurrence of negative and positive events helps clarify the risk and protective mechanisms associated with depression, thereby informing its treatment and prevention.

**Figure 1:**
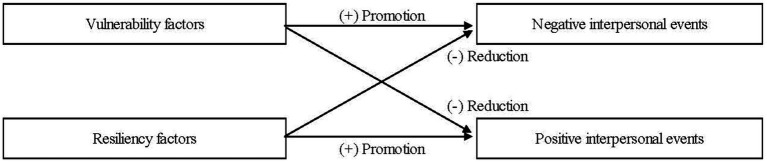
Four processes in interpersonal event generation.

Theoretical and empirical studies on stress generation suggest four different event-generation processes, shown in Figure 1. To date, few studies have tested these four processes simultaneously within a single analytic model. This study was designed to address this gap in the literature by examining whether vulnerable (socially withdrawn and excessive reassurance-seeking) and resilient (prosocial) behaviors uniquely and differentially predict the occurrence of negative and positive interpersonal events among young adolescents.

### 1.1. Negative and positive life events as risk and protective factors for depression

Negative life events concern individuals’ life experiences that are objectively rated as threatening and unpleasant (i.e., stressful), such as interpersonal rejection and loss. Previous studies have consistently found that negative life events are an important risk factor for depression (Hammen, 2005; Liu and Alloy, 2010). Research has established that life stress precedes depression symptoms, prospectively predicting them across the life span (Vrshek-Schallhorn et al., 2020).

Recent studies have advanced previous research and yielded significant findings concerning the relationship between life events and depression. First, they have provided novel ideas concerning the *direction* of the relationship between negative life events (environment) and depression (person). Previous studies have viewed individuals as passive respondents to their environment and have shown a unidirectional effect of life events on depression (the stress exposure model of depression). In contrast, recent studies using a transactional approach have viewed individuals as active agents in shaping their environment and revealed a bidirectional relationship between life events and depression (Hammen, 1991; Liu and Alloy, 2010). These studies indicate that depressed and depression-vulnerable individuals actively contribute to the occurrence of negative life events, thereby developing, maintaining, or recurring depression.

Second, recent studies have indicated that not only negative but also *positive* life events play an important role in the etiology of depression. Researchers have suggested that the presence of positive interpersonal events protects individuals against depression, whereas their absence leads them to become depressed (e.g., Lewinsohn, 1974). Empirical studies support this idea (e.g., Haeffel and Vargas, 2011; Herres and Kobak, 2015). For example, studies have found that greater social support protects individuals against depression, whereas deficits in social support lead to higher levels of depressive symptoms (Ibarra-Rovillard and Kuiper, 2011). These findings are reasonable because positive interpersonal experiences, such as perceiving social support and acceptance from others, serve to fulfill individuals’ fundamental needs to be connected with others and social groups (Ibarra-Rovillard and Kuiper, 2011).

Identifying the predictors of both negative and positive events helps to better understand the mechanisms associated with depression and to inform its treatment and prevention. Such predictors have been examined and identified through research on the stress generation model.

### 1.2. Stress generation research and four patterns of event generation

The stress generation model was proposed by Hammen (1991, 2006). This model conceptualizes stress as environmental and objective *stressors*. These stressors are defined as changes or conditions in the environment that individuals actually experience, such as the occurrence of a negative life event. The stressors are distinguished from two other key components of stress: stress appraisals (i.e., the individuals’ subjective evaluations of how the event is threatening, challenging, or meaningful) and stress responses (i.e., the individuals’ psychological and physiological reactions to either the event itself or to their appraisals of the event) (Cohen et al., 1997; Grant et al., 2003).

The stress generation model postulates that depressed and depression-prone individuals create their stressful events (i.e., negative stressors) that lead to the onset, maintenance, or recurrence of depression. The stress generation model views stressful events as dependent (defined as stressful events that are at least in part dependent on the behaviors of an individual [e.g., interpersonal conflicts]) or independent (defined as stressful events that are independent of an individual’s behaviors [e.g., sickness of others]). According to this model, depressed and depression-prone individuals generate *dependent* and *interpersonal* events (Hammen, 1991, 2006). This model could explain the mechanisms of not only the recurrence (Hammen, 1991, 2005) but also the onset, maintenance, and exacerbation of depression (Liu and Alloy, 2010).

While initial research has shown that depressive symptoms contribute to stress generation, recent studies have increasingly examined the contribution of individual characteristics other than depressive symptoms. Studies have shown that various vulnerability factors for depression increase the occurrence of dependent and interpersonal negative events. These factors include personal vulnerabilities, such as negative cognitive styles and neuroticism, and interpersonal vulnerabilities, such as anxious attachment styles and social behavioral deficits (for reviews, see Hammen, 2005, 2006; Liu and Alloy, 2010; Liu, 2013).

More recently, researchers have expanded the original idea of the stress generation model and provided novel findings. There are two lines of research in this area. The first line has focused on *positive interpersonal events* and examined whether vulnerability factors *decrease* their occurrence. Empirical studies have found that social behavioral deficits (Lewinsohn, 1974), self-criticism (Shahar and Priel, 2003), and trait negative affect (Hamilton et al., 2017) predict fewer positive events.

The second line of research has focused on *resiliency factors* and examined whether they *decrease* the occurrence of negative events or *increase* the occurrence of positive events. Empirical studies have found that enhancing attributional styles predict fewer negative events (Kleiman et al., 2013), whereas extraversion (Magnus et al., 1993), dependency (Shahar and Priel, 2003), trait positive affect (Hamilton et al., 2017), gratitude and meaning in life (Disabato et al., 2017), and prosocial behavior (Kuroda and Sakurai, 2003) predict more positive events.

Given these findings, we can posit the four event generation processes shown in Figure 1 (see Liu and Alloy, 2010 for a similar discussion; and Kuroda and Sakurai, 2003; Shahar and Priel, 2003; Hamilton et al., 2017, for related findings). Figure 1 shows that vulnerability factors increase negative events and decrease positive events, whereas resiliency factors decrease negative events and increase positive events. Increased negative events and decreased positive events have distinct implications for individuals, but both put them at risk for depression. Decreased negative events and increased positive events also have different meanings for individuals, but both protect them against depression. These four situations are different, but each plays an important role in the mechanisms for depression. Therefore, it is important to determine the predictors of these situations. It is particularly significant to identify vulnerability factors that can simultaneously predict *both* increased negative events and decreased positive events and resiliency factors that can simultaneously predict *both* decreased negative events and increased positive events, because the former factors have a double risk for depression, and the latter factors have a double buffer against depression. However, to date, few studies have focused on such factors to test four event generation processes simultaneously within a single analytic model (see Kuroda and Sakurai, 2003; Shahar and Priel, 2003; Hamilton et al., 2017, for related studies that examined some of these processes). This study addressed this gap by examining vulnerable and resilient interpersonal behaviors as an event-generating factor and young adolescents as a study sample.

### 1.3. Importance of examining interpersonal behaviors and young adolescents

Stress generation research has examined various stress-generating factors and age samples (Hammen, 2006; Liu and Alloy, 2010; Liu, 2013; Santee et al., 2023). Of these factors and samples, this study focused on interpersonal behaviors and young adolescents for the following reasons.

Interpersonal behaviors are theoretically and clinically important predictors of stress generation (e.g., Coyne, 1976; Hammen, 2006; Rudolph, 2009; Liu, 2013). Specifically, they are the most proximal (direct) and potent predictors of stress generation (Rudolph, 2009; Liu, 2013), and thus, are critical targets for preventing negative event generation. Interpersonal behaviors also mediate the effects of personality factors (e.g., negative inferential styles and attachment styles) on event generation (Liu and Alloy, 2010; Liu, 2013; Santee et al., 2023). Although personality is more fixed and difficult to change, behavior is comparatively flexible and easier to change. The changeable nature of behavior increases the feasibility and effectiveness of intervention and prevention.

Early adolescence is a critical developmental period associated with increased vulnerability to depression. Research has found that depression increases sharply from childhood to adolescence, and depression that occurs during adolescence continues into the later period (Avenevoli et al., 2008). Hence, it is crucial to identify the vulnerability and resiliency factors of depression during early adolescence. Research has also found that interpersonal contexts are deeply involved in depression among the youth (Rudolph et al., 2008; Rudolph, 2009; Schwartz-Mette et al., 2020). These findings highlight the necessity of examining youth vulnerability and resiliency in interpersonal contexts. The stress generation model provides a promising framework for such examinations.

### 1.4. Relationship between interpersonal behaviors and negative and positive interpersonal events among youth

As discussed above, it is significant to examine vulnerability factors that could simultaneously predict *both* increased negative events and decreased positive events and resiliency factors that could simultaneously predict *both* decreased negative events and increased positive events. Theory and research concerning youth’s interpersonal stress generation have postulated that social withdrawal and excessive reassurance-seeking (vulnerability factors) and prosocial behavior (resiliency factor) are potential predictors of both negative and positive interpersonal events (Prinstein et al., 2005; Zimmer-Gembeck et al., 2007; Rudolph et al., 2008; Rudolph, 2009; Shih et al., 2009). As elaborated in subsequent sections, these three behaviors exhibit differences along the dimensions of approach-avoidance and adaptive-maladaptive. These three behaviors have a strong theoretical background and have been examined in relation to negative and positive events within peer relationships.

Social withdrawal is a form of avoidant behavior defined as the tendency to consistently avoid familiar and unfamiliar peers (Bowker et al., 2021). Behavioral theories of depression postulate that avoidant behaviors reduce rewards and positive reinforcers from the social environment, thereby developing and exacerbating depression (e.g., Lewinsohn, 1974). Recent interpersonal theories of youth depression posit that social withdrawal reduces positive social interactions while also promoting negative interpersonal outcomes (Rudolph et al., 2008; Rudolph, 2009). Consistent with these theoretical assumptions, empirical research has shown that socially withdrawn youth are likely to experience both less positive peer events, such as peer acceptance (e.g., Zimmer-Gembeck et al., 2007; Barzeva et al., 2020), and more negative peer events, such as peer rejection, exclusion, and victimization (for a review, see Ladd et al., 2021).

Excessive reassurance-seeking is central to Coyne’s interpersonal theory of depression (Coyne, 1976; Joiner et al., 1999) and is defined as individuals’ tendency to persistently seek assurance from others about whether others truly care about them (Joiner et al., 1992). This behavioral tendency irritates others, elicits rejection, and ultimately leads to depression (Coyne, 1976; Joiner et al., 1999). A meta-analysis indicated a cross-sectional relationship between excessive reassurance-seeking and interpersonal rejection (Starr and Davila, 2008). A recent longitudinal study by Stroud et al. (2018) found that excessive reassurance-seeking predicts acute and chronic interpersonal stress over time among early adolescent girls (see Prinstein et al., 2005 for an exception). Research also found that (self-reported) excessive reassurance-seeking predicts lower levels of (friend-reported) positive friendship quality over time among girls but not among boys (Prinstein et al., 2005).

Prosocial behaviors are defined as voluntary behaviors intended to benefit others, such as helping, caring, and sharing (Eisenberg et al., 2006). They serve as protective factors against depression (for a meta-analytic review, see Memmott-Elison et al., 2020). Social-developmental theorists posit that children and adolescents’ prosocial behaviors play a vital role in forming, maintaining, and enhancing positive relationships with their peers (e.g., Dirks et al., 2018). The social exchange theory proposes that the norm of prosocial reciprocity leads people who receive prosocial behavior from others to reciprocate by exhibiting the same beneficial behavior (Flynn and Yu, 2021). This suggests that individuals who engage in prosocial behaviors are more likely to receive positive responses and less likely to receive negative responses from others. Consistent with these theoretical assumptions and suggestions, empirical studies have found that the youth’s prosocial behaviors show negative associations with adverse interpersonal events, such as peer rejection and victimization (e.g., Zimmer-Gembeck et al., 2007; Sugimura et al., 2017; Di Giunta et al., 2018), and positive associations with positive interpersonal outcomes, such as peer acceptance (e.g., Jin et al., 2022; for reviews, see Wentzel, 2014; Dirks et al., 2018).

### 1.5. Study purpose and hypotheses

Previous studies have shown that social withdrawal and excessive reassurance-seeking predict more negative and fewer positive peer events, whereas prosocial behavior predicts more positive and fewer negative peer events. However, no study has measured all these variables in the same sample and examined their relationships simultaneously within a single analytic model. This examination is critical to determine whether vulnerable (socially withdrawn and reassurance-seeking) and resilient (prosocial) behaviors predict negative and positive peer events in unique and different ways. If each behavior uniquely predicts *both* events, we could regard it as a critical risk or protective factor for depression and as an important target for preventing depression.

This study aimed to address this important gap in the literature on interpersonal stress generation. The hypotheses were formulated based on the following rationale. Social withdrawal (e.g., Lewinsohn, 1974; Trew, 2011), excessive reassurance-seeking (e.g., Coyne, 1976; Joiner et al., 1999), and prosocial behaviors (e.g., Eisenberg et al., 2006; Memmott-Elison et al., 2020) stem from different theoretical foundations and are conceptually and functionally distinct. These three behaviors exhibit differences along the dimensions of approach-avoidance and adaptive-maladaptive. Specifically, social withdrawal and excessive reassurance-seeking are both forms of maladaptive behaviors. However, social withdrawal is characterized by *avoidance of others* (Bowker et al., 2021), whereas excessive reassurance-seeking manifests as a *maladaptive approach toward others* (Pettit and Joiner, 2006). Consequently, although both behaviors are expected to result in fewer positive, and more negative, interpersonal events, they do so through unique mechanisms.

In contrast, prosocial behaviors reflect adaptive behaviors. Importantly, adaptive (or resilient) behaviors are not simply the polar opposites of maladaptive (or vulnerable) behaviors (Santee et al., 2023). More specifically, higher levels of prosocial behaviors do not necessarily indicate lower levels of social withdrawal or excessive reassurance-seeking, nor do lower levels of prosocial behaviors imply higher levels of social withdrawal or excessive reassurance-seeking. Prosocial behaviors involve an *adaptive approach toward others* (Curry et al., 2018; Hui et al., 2020), and therefore differ in dimension from social withdrawal or excessive reassurance-seeking. Thus, prosocial behaviors uniquely predict positive and negative interpersonal events above and beyond social withdrawal and excessive reassurance-seeking.

Based on these discussions, the following hypotheses were formulated:

*Hypothesis 1:* More social withdrawal uniquely predicts a higher occurrence of negative peer events (Hypothesis 1-1) and a lower occurrence of positive peer events (Hypothesis 1-2).

*Hypothesis 2:* Excessive reassurance-seeking uniquely predicts a higher occurrence of negative peer events (Hypothesis 2-1) and a lower occurrence of positive peer events (Hypothesis 2-2).

*Hypothesis 3:* More prosocial behavior uniquely predicts a lower occurrence of negative peer events (Hypothesis 3-1) and a higher occurrence of positive peer events (Hypothesis 3-2).

In testing the above hypotheses, the following two points were considered: First, previous studies have found that depressive symptoms are associated with three interpersonal behaviors and negative and positive interpersonal events. Therefore, this study controlled for depressive symptoms in testing the hypotheses. Second, previous studies examined possible sex differences in the relationships between interpersonal behaviors and negative and positive peer events. However, the findings were inconsistent for social withdrawal (see Rubin et al., 2010 for a review) and prosocial behavior (see Dirks et al., 2018 for a review), and were lacking regarding excessive reassurance-seeking. Thus, this study made no specific predictions concerning sex differences and examined them on an exploratory basis.

## 2. Materials and methods

### 2.1. Participants

Participants were seventh (12–13 years old), eighth (13–14 years old), and ninth graders (14–15 years old) recruited from a public middle school in Japan. All participants were Japanese and had the same cultural background.

Of the 202 students who participated in this study, four provided incomplete data. Thus, the final sample consisted of 198 students (109 girls; 106 seventh graders, 29 eighth graders, and 63 ninth graders).

### 2.2. Measures

#### 2.2.1. Social withdrawal

Social withdrawal was measured using the behavior-to-participate-in-peer-relations subscale (seven items) of the Social Skills Scale in School (SSS-S; Togasaki and Sakano, 1997). This subscale assesses students’ behavioral tendencies to withdraw from their peers. To minimize participant time and effort burden, this study used five items that showed the highest factor loadings in Togasaki and Sakano’s, 1997 scale construction study. Sample items include “I play alone, away from my peers,” “I try to join in with my peers who are playing, but it is hard for me to do so,” and “I often stare at my peers playing.” Participants rated the frequency of each behavior on a 4-point Likert scale ranging from 1 (*not at all*) to 4 (*always*). A higher score indicates a higher level of social withdrawal. Togasaki and Sakano (1997) demonstrated that the SSS-S had sufficient reliability and validity. In the present study, Cronbach’s alpha coefficient for internal consistency was 0.80.

#### 2.2.2. Reassurance-seeking

A reassurance-seeking scale for young adolescents had not been developed in Japan when this study was conducted. Thus, we developed a new scale based on the definition of reassurance-seeking and the Japanese-translated version (Katsuya, 2001) of the Depressive Interpersonal Relationships Inventory-Reassurance-Seeking Subscale (Joiner et al., 1992). The new scale consisted of four items (e.g., “I seek reassurance from my friends as to whether they really care about me,” “I ask my friends how they truly feel about me,” and “I ask my friends if they think badly of me”) and asked the participants to rate the frequency of each behavior on a 4-point Likert scale ranging from 1 (*not at all*) to 4 (*always*). A higher score indicates a higher level of reassurance-seeking. Cronbach’s alpha coefficient was 0.83 in this study.

#### 2.2.3. Prosocial behavior

Prosocial behaviors were assessed using the empathic and helping behavior subscale (six items) of the Social Skills Scale (SSS; Shoji, 1991). To minimize participant burden, this study used five items that showed the highest factor loadings in Shoji’s (1991) scale construction study. Sample items included “I encourage and console my friends when they fail,” “I help my friends when they are in trouble,” and “When a friend looks lonely and alone, I call out to him or her.” Participants rated the frequency of each behavior on a 4-point Likert scale ranging from 1 (*not at all*) to 4 (*always*). A higher score indicates a higher level of prosocial behavior. Shoji (1991) demonstrated that the SSS had sufficient reliability and validity. In the present study, Cronbach’s alpha coefficient for internal consistency was 0.80.

#### 2.2.4. Negative and positive peer events

The shortened version of the Negative and Positive Life Events in Interpersonal Domain Scale (Takahira, 1998) was used to measure the frequencies of negative and positive peer events. This scale assesses the frequencies of negative and positive life events that occur in relationships with friends, romantic partners, and family members. As this study focused on peer relationships, the scale was modified to assess events within such relationships. In addition, because the stress generation model posits that individuals generate *dependent* interpersonal events, this study removed two items concerning *independent* interpersonal events (“My close friend got sick or injured” and “I met my old friend by chance”) from the original scale. The modified version consisted of 13 items for negative (e.g., “I was ignored by my friend,” “I was misunderstood by my friend,” and “I was criticized by my friend”) and 13 items for positive (e.g., “I was trusted by my friend,” “My friend was kind to me,” and “My friend helped me”) peer events. This study used a 4-point Likert scale ranging from 0 (*did not happen at all*) to 3 (*frequently happened*) to assess the frequency of each event.

#### 2.2.5. Depressive symptoms

Depressive symptoms were measured using the Child Depression Inventory-Japanese Short Version (CDI-JSV; Kuroda and Sakurai, 2011). The original inventory (CDI), developed by Kovacs (1983), is a widely used measure for assessing depressive symptoms in early adolescents. Each item contained three statements (e.g., “I am sad once in a while,” “I am sad many times,” and “I am sad all the time”), and participants were asked to choose the statement that best described their level of depressive symptoms during the past two weeks. The score for each item ranges from 1 to 3, with a higher score indicating greater symptom severity. Previous studies have shown that this scale has sufficient internal consistency and construct validity (Kuroda and Sakurai, 2003, 2011). In this study, Cronbach’s alpha coefficient was 0.90.

### 2.3. Procedure

When this study was conducted (2002), an institutional ethics review board had not yet been established in most faculties of psychology in Japan, including our affiliation. Therefore, to ensure the meeting of the required ethical standards, we reviewed this study’s contents (i.e., purpose, questionnaire, procedure, and design) carefully, in consultation with fellow peer researchers and the participating school principal and teachers. The questionnaire was anonymous; therefore, each student could not be identified based on their responses.

The final consent to conduct this study and participation of the students was obtained from the school principal. In the majority of Japanese schools, including the school in this study, the parents of the children gave the school principal the authority to decide whether to allow their children to participate in the research study.

The classroom teachers explained to their students the purpose and outline of the study, handed them a package containing all the measures, and guided them to complete the questionnaires. All students were assured of confidentiality and anonymity and agreed to participate in the study.

### 2.4. Data analysis

We conducted a multiple-group path analysis, which allowed us to evaluate the hypothesized model and sex differences in the parameters of the model (Kline, 2011; Lefcheck, 2021). This analysis consisted of three steps. First, we compared a fully unconstrained model (a baseline model where all parameters were allowed to vary across sexes) and a fully constrained model (a comparison model where all parameters were set to be equal across the sexes) using a *χ*^2^ difference test. Second, we constrained one parameter at a time to be equal across the sexes and compared the constrained model to the fully unconstrained model using the *χ*^2^ difference test. If a significant difference was found, we could assume that the parameter that we imposed on the constraint would differ between the sexes. Third, we created a partially constrained model—in which the parameters that differed between the sexes were allowed to vary, and the ones that did not were set to be equal—and compared this model to the fully unconstrained model using the *χ*^2^ difference test. If a significant difference was not found (i.e., the fit of the partially constrained model was equal to that of the fully unconstrained baseline model), we could accept the partially constrained model. We also evaluated the fit of the models using the Comparative Fit Index (CFI), Incremental Fit Index (IFI), and Root Mean Square Error of Approximation (RMSEA).

The parameters in the models were determined by maximum likelihood estimation. The AMOS software package (version 19.0) was used for a multiple-group path analysis. SPSS 19.0 was used to calculate descriptive statistics and correlations between variables.

## 3. Results

### 3.1. Means, standard deviations, and intercorrelations of the measures

Table 1 presents the means and standard deviations of all measures according to sex. To examine sex differences in the measures, we first performed a multivariate analysis of variance. However, Box’s M test for homogeneity of covariance matrices was significant (*p* < 0.001). Therefore, we performed Welch’s independent samples *t*-tests (two-tailed), which do not require homogeneity of variance and are used when sample sizes differ across groups. The results showed that girls scored higher than boys on excessive reassurance-seeking (*t* (193) = 2.10, *p* < 0.05, Cohen’s *d* = 0.30), prosocial behavior (*t* (156) = 6.46, *p* < 0.001, Cohen’s *d* = 0.92), and positive peer events (*t* (181) = 6.57, *p* < 0.001, Cohen’s *d* = 0.94).

**Table 1:** Descriptive statistics and correlations between measures.

		1		2		3		4		5		6	
1	Social withdrawal			0.25*		−0.20		0.55**		−0.37**		0.50**	
2	Excessive reassurance-seeking	0.16				0.13		0.19		0.01		0.04	
3	Prosocial behavior	0.02		0.25*				−0.22*		0.50**		−0.33**	
4	Negative peer events	0.26**		0.24*		0.20*				−0.10		0.55**	
5	Positive peer events	−0.30**		0.27**		0.40**		0.28**				−0.53**	
6	Depressive symptoms	0.65**		0.11		0.01		0.36**		−0.35**			
	*M* (*SD*) for girls	8.09 (3.15)		6.68 (2.65)		16.50 (2.04)		10.80 (6.87)		25.36 (6.77)		18.76 (5.10)	
	*M* (*SD*) for boys	8.00 (2.87)		6.00 (1.89)		14.19 (2.83)		9.80 (7.06)		18.67 (7.40)		18.34 (5.22)	

* *p* < 0.05, ** *p* < 0.01 (two-tailed tests). Correlations above the diagonal are for boys, and those below the diagonal are for girls. Ms reflect mean scale scores.

Table 1 also shows the correlations between the study measures by sex. For girls, three interpersonal behaviors correlated with negative and positive peer events in the predicted directions, with two exceptions (i.e., positive correlations between reassurance-seeking and positive peer events, and between prosocial behavior and negative peer events). For boys, similar predicted correlations were found except for no correlation between reassurance-seeking and positive peer events. However, these expected and unexpected correlations might occur because of the correlations between the three interpersonal behaviors and between negative and positive peer events. This issue could be resolved by performing a path analysis, as discussed below.

### 3.2. Test of hypotheses

Before testing the hypotheses, the issues of collinearity were checked by examining the variance inflation factor (VIF). The VIF values for all independent and controlled variables ranged between 1.06 and 1.76, well below the threshold of 10.00 (Kline, 2011), indicating no collinearity issues.

A multiple-group path analysis was performed to test the hypotheses and possible sex differences. The fully unconstrained model was saturated and thus showed a perfect fit to the data. The fully constrained model fitted the data well (*χ^2^* (15) = 29.52, *p* < 0.05, CFI = 0.95, IFI = 0.95, RMSEA = 0.07). The *χ*^2^ difference test showed a significant difference between these two models (*Δχ^2^* (15) = 29.52, *p* < 0.05), indicating that the fully constrained model fitted the data worse than the fully unconstrained model did. This suggests that one or more of the parameters would vary across the sexes.

To determine which parameters differed between the sexes, we constrained one parameter at a time to be equal across the sexes and compared the constrained model to the fully unconstrained one. Significant *χ*^2^ differences were found between the fully unconstrained model and (1) the constrained model in which the path from social withdrawal to negative peer events was set to be equal (*Δχ^2^* (1) = 5.41, *p* < 0.05); and (2) the constrained model in which the covariance between depression and prosocial behaviors was set to be equal (*Δχ^2^* (1) = 7.19, *p* < 0.01). These results suggest that the path and covariance differed between the sexes. Thus, we created a partially constrained model in which these two parameters were allowed to vary across sex and the other parameters were set to be equal.

The partially constrained model fitted the data very well (*χ^2^* (13) = 12.63, *p* = 0.48, CFI = 1.00, IFI = 1.00, RMSEA = 0.00). The *χ*^2^ difference test showed no significant difference between the partially constrained and fully unconstrained models (*Δχ*^2^ (13) = 12.63, *p* = 0.48), suggesting that their fits were equivalent. Therefore, we accepted the partially constrained model because it would be equally informative as, but more parsimonious than, the fully unconstrained model.

**Figure 2:**
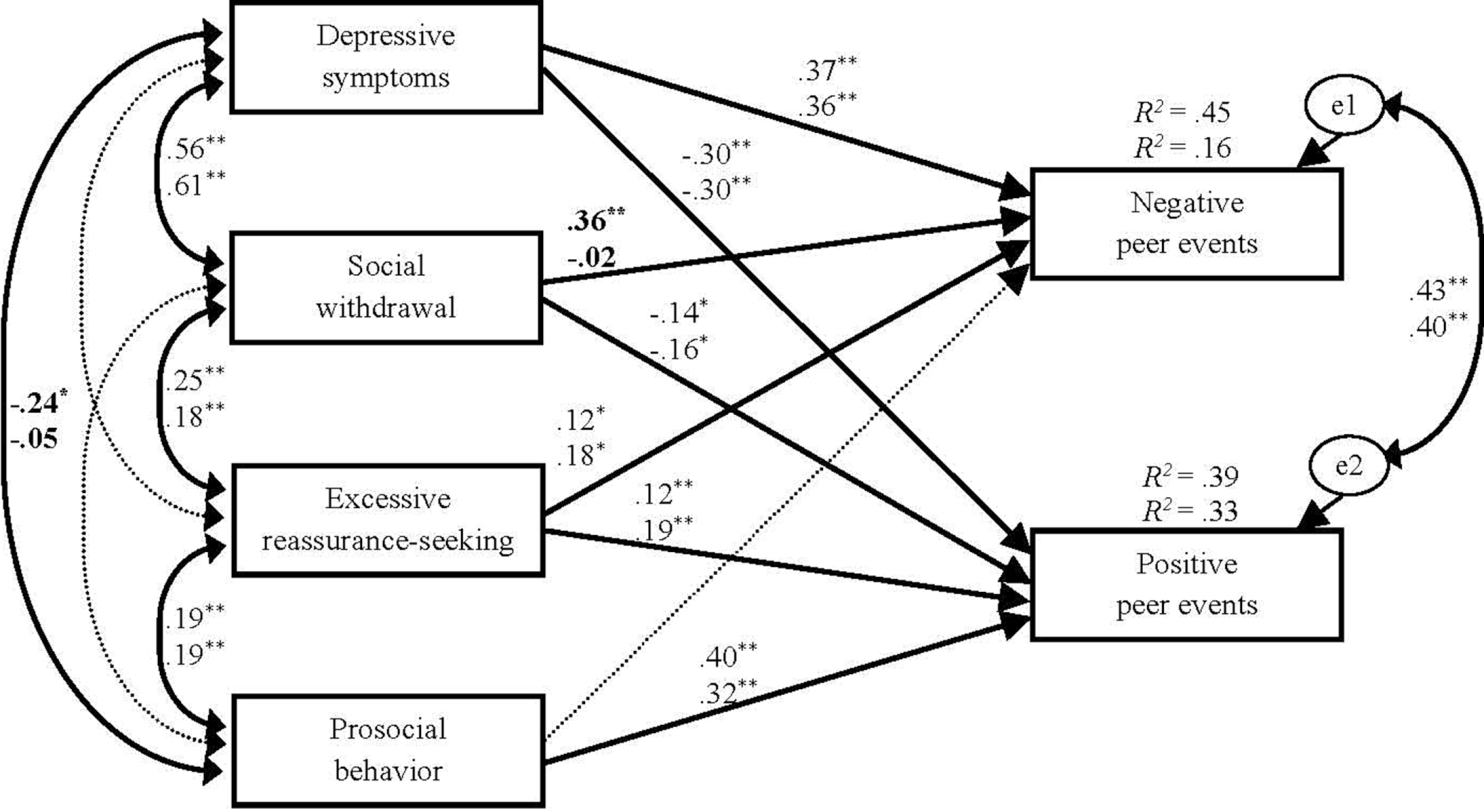
Results of multiple-group path analysis. The values at the top are for boys, and those at the bottom are for girls. The bold values indicate significant differences between the sexes. Non-significant paths and covariances are shown in dashed lines. * *p* < 0.05, ** *p* < 0.01 (two-tailed).

Figure 2 shows the path coefficients and covariances of the partially constrained model. For boys, social withdrawal and excessive reassurance-seeking predicted more negative peer events (consistent with hypotheses 1–1 and 2–1), social withdrawal predicted fewer positive peer events (consistent with hypothesis 1-2), and prosocial behavior predicted more positive peer events (consistent with hypothesis 3-2). However, excessive reassurance-seeking predicted more positive peer events (contrary to hypothesis 2-2), and prosocial behavior was not a significant predictor of negative peer events (inconsistent with hypothesis 3-1). For girls, the same results were obtained, except for a non-significant path from social withdrawal to negative peer events (inconsistent with hypothesis 1-1). For both sexes, depressive symptoms predicted more negative and fewer positive peer events.

## 4. Discussion

This study tested four processes in interpersonal stress generation by examining whether vulnerable and resilient interpersonal behaviors uniquely predict negative and positive peer events among youth. The results were consistent with the hypotheses, with a few exceptions.

Social withdrawal predicted fewer positive peer events for both boys and girls (supporting hypothesis 1-2). It also predicted more negative peer events for boys (supporting hypothesis 1-1), but not for girls. The latter sex-specific result can be explained in terms of gender role norms. Previous studies suggest that social withdrawal violates male gender roles but not female gender roles; thus, socially withdrawn boys are more likely than socially withdrawn girls to receive negative responses from peers (Doey et al., 2014; Sugimura et al., 2017). Our findings are consistent with these studies, suggesting that girls’ social withdrawal has a less negative impact on peer relationships than that of boys. Boys’ social withdrawal is a serious problem because it predicts both fewer positive peer events and more negative peer events.

Excessive reassurance-seeking predicted both more negative peer events (consistent with hypothesis 2-1) and more positive peer events (contrary to hypothesis 2-2), for boys and girls. We suggest the following possibilities for this finding: excessive reassurance-seeking might have positive effects in the short term but negative effects in the long run. Specifically, reassurance seekers could initially elicit positive reactions from their partners because the partners try to relieve reassurance seekers’ concerns and anxiety. These positive effects might be more likely for younger people because excessive reassurance-seeking is more common and thus likely perceived as less norm-violating among children and adolescents (Starr and Davila, 2008). However, reassurance seekers are finally rejected by their partners because they persistently seek assurance, even though the partners repeatedly provide it for them (Coyne, 1976). The present findings may reflect the different effects of reassurance-seeking over time.

Prosocial behavior predicted more positive peer events for boys and girls (supporting hypothesis 3-2). This result was obtained even after controlling for vulnerable interpersonal behaviors and depressive symptoms, suggesting that prosocial behavior might be a promising resilience factor that predicts positive interpersonal outcomes. The social exchange theory (e.g., Flynn and Yu, 2021) helps explain why prosocial behavior contributes to positive interpersonal outcomes. The theory posits that people hold the social norm that if they receive prosocial behavior from an individual, they should reciprocate by exhibiting the same beneficial behaviors. Owing to this norm concerning prosocial reciprocity, youths who are more likely to engage in prosocial behaviors toward their peers are more likely to receive positive responses from their peers in return for their behaviors. However, inconsistent with hypothesis 3-1, prosocial behavior was not a significant predictor of negative peer events for boys and girls. While previous studies have found that prosocial behaviors reduce negative interpersonal outcomes (e.g., Di Giunta et al., 2018), several recent findings suggest that the effects differ depending on the subtypes of prosocial behavior. For example, Findley-Van Nostrand and Ojanen (2018) distinguished between altruistic (beneficial to others without expectation of personal gain) and proactive (instrumental, self-benefiting) behaviors and examined their associations with peer rejection. They found that altruistic prosocial behavior was negatively associated with peer rejection, whereas proactive prosocial behavior was positively associated with peer rejection. The prosocial behaviors examined in this study might include different subtypes, which might result in a null association between prosocial behavior and negative peer events.

The present results suggest that no sex differences exist in the paths from the three interpersonal behaviors to negative and positive peer events, except for the path from social withdrawal to negative peer events. This finding is consistent with recent meta-analytic results that showed no sex differences in the associations between withdrawal and positive friendships (Dryburgh et al., 2022), prosocial behavior and positive friendships (Dryburgh et al., 2022), and excessive reassurance-seeking and interpersonal rejection (Starr and Davila, 2008) among the youth. The contribution of these three interpersonal behaviors to negative and positive interpersonal events might be relatively similar across the sexes.

This study has important theoretical and clinical implications. Theoretically, this study integrated previous studies to posit the four event-generation processes, as depicted in Figure 1 (see also Liu and Alloy, 2010 for a similar discussion). We examined these processes simultaneously within a single analytic model to provide empirical evidence for three of the four processes. Specifically, 1) vulnerable factors (excessive reassurance-seeking and boys’ social withdrawal) uniquely predicted increased negative peer events; 2) vulnerable factor (social withdrawal) uniquely predicted decreased positive peer events; and 3) resilient (prosocial) factor uniquely predicted increased positive peer events. These findings demonstrate that vulnerable and resilient interpersonal behaviors uniquely and differentially predict negative and/or positive interpersonal events. This highlights the significance of considering both vulnerability and resiliency factors and both negative and positive events in stress generation research.

Clinically, this study suggests that treatment and prevention might focus on both reducing youths’ vulnerability and cultivating their resiliency to prevent negative interpersonal experiences and promote positive interpersonal experiences. Given the present findings, it is necessary to reduce social withdrawal and excessive reassurance-seeking and cultivate prosocial behavior among young adolescents. Reducing boys’ social withdrawal would be particularly important, as it predicted *both* increased negative peer events and decreased positive peer events.

This study has several limitations. First, because the study design was cross-sectional and correlational, we have limited ability to judge the causality of the observed relationships. Longitudinal studies are necessary to provide reasonable evidence for the causal direction. Second, this study relied on self-reported scales, which are subject to response bias. One important factor that yields a response bias is the negative mood of the respondents. This study controlled for participants’ depressive symptoms, thereby reducing the mood-congruent biases that likely occur when they rate interpersonal behaviors and negative and positive peer events. However, we cannot reject other possibilities of response bias (e.g., shared method variance). It is impossible to rule out response bias entirely, which is an inherent limitation of studies using self-reported measures. Future studies should include more objective measurement methods, such as observational methods for interpersonal behaviors and interview methods for peer-related events. Finally, because this study examined a community sample of adolescents, it is unclear whether the findings can be generalized to clinical samples. It is necessary to replicate the findings of this study by examining clinical or high-risk samples of young adolescents.

## 5. Conclusion

This study found that vulnerable (social withdrawal and excessive reassurance-seeking) and resilient (prosocial behaviors) interpersonal behaviors uniquely and differentially predicted negative and positive peer events among young adolescents. The findings underscore the significance of considering both vulnerability and resiliency factors and both negative and positive events in stress generation research. Additionally, the findings suggest that not only reducing interpersonal vulnerability (social withdrawal and excessive reassurance-seeking) but also cultivating interpersonal resiliency (prosocial behavior) might be helpful for the treatment and prevention of depression, and the promotion and maintenance of well-being among young adolescents. Future studies would benefit from replicating and extending the present findings by adopting a longitudinal design and interview method, and by examining vulnerability and resiliency factors, interpersonal contexts, and age samples that differ from those of this study.

## Data availability statement

The raw data supporting the conclusions of this article will be made available by the authors, without undue reservation.

## Ethics statement

Ethical approval was not required for the study involving humans in accordance with the local legislation and institutional requirements. Written informed consent to participate in this study was not required from the participants or the participants' legal guardians/next of kin in accordance with the national legislation and the institutional requirements.

## Author contributions

The author confirms being the sole contributor of this work and has approved it for publication.
